# MP Resulting in Autophagic Cell Death of Microglia through Zinc Changes against Spinal Cord Injury

**DOI:** 10.1155/2016/6090316

**Published:** 2016-01-06

**Authors:** Dingding Li, Guannan Wang, Donghe Han, Jing Bi, Chenyuan Li, Hongyu Wang, Zhiyuan Liu, Wei Gao, Kai Gao, Tianchen Yao, Zhanghui Wan, Haihong Li, Xifan Mei

**Affiliations:** ^1^Department of Orthopedic Surgery, First Affiliated Hospital of Liaoning Medical University, Jinzhou 121000, China; ^2^Department of Chemistry, College of Pharmacy, Liaoning Medical University, Jinzhou 121000, China; ^3^Department of Neurobiology, Key Laboratory of Neurodegenerative Diseases of Liaoning Province, Liaoning Medical University, Jinzhou 121000, China; ^4^Department of Basic Medical Sciences, Liaoning Medical University, Jinzhou 121000, China

## Abstract

Methylprednisolone pulse therapy (MPPT), as a public recognized therapy of spinal cord injury (SCI), is doubted recently, and the exact mechanism of MP on SCI is unclear. This study sought to investigate the exact effect of MP on SCI. We examined the effect of MP in a model of SCI in vivo and an LPS induced model in vitro. We found that administration of MP produced an increase in the Basso, Beattie, and Bresnahan scores and motor neurons counts of injured rats. Besides the number of activated microglia was apparently reduced by MP in vivo, and Beclin-1 dependent autophagic cell death of microglia was induced by MP in LPS induced model. At the same time, MP increases cellular zinc concentration and level of ZIP8, and TPEN could revert effect of MP on autophagic cell death of microglia. Finally, we have found that MP could inhibit NF-*κβ* in LPS induced model. These results show that the MP could result in autophagic cell death of microglia, which mainly depends on increasing cellular labile zinc, and may be associated with inhibition of NF-*κβ*, and that MP can produce neuroprotective effect in SCI.

## 1. Introduction

Spinal cord injury (SCI) has been studied for over 100 years, and its harm that lies in causing lifelong disability and psychological burden have been described in considerable papers [1]. However, exactly effective therapies of SCI have not advanced to improve the recovery of SCI patients. Therefore, it is critical to find out therapeutic strategies for SCI patients [2]. The pathophysiology of SCI involves two mechanisms, primary and secondary mechanisms [3]. Secondary injury mechanisms, which are more pivotal in the recovery of SCI, include inflammation, oxidization, immunological reaction, electrolyte disorder, vascular damage, and loss of energy balance [4].

Methylprednisolone (MP) is a synthetic glucocorticoid agonist, with major properties of potent anti-inflammation and suppressing immunity, and methylprednisolone pulse therapy (MPPT) is the only public recognized therapy of SCI at present in acute phase, in order to minimize neurological damage [5]. Besides, MP gets rid of free radical-induced or iron-catalyzed lipid peroxidation and protein oxidative damage [6, 7]. MP also has protective effect on vascular injury after SCI, through diverse aspects of tissue edema, vascular permeability, and polymorphonuclear cell infiltration [8]. However, the exact mechanism of MP on SCI is not perceived for its complicated mechanisms. And there are new researches on high dose of MP, giving out contrary results against the protection of MP on SCI [9, 10].

Autophagy is involved not only in the protein synthesis and degradation, digestion of intracellular components, but also in the execution of cell death, nonapoptotic programmed cell death, which is also known as autophagic cell death [11]. Previous studies have reported that autophagic cell death occurs in various diseases. Autophagic cell death was induced through glutamate-mediated GSK-3*β* activation in astrocytes [12] or through elevation of Beclin-1 in neurons [13] after traumatic brain injury. Autophagic cell death participates in cardiac myocytes during myocardial infarction, ischemia/reperfusion, and heart failure [14]. Autophagic cell death of hepatocyte results in liver graft dysfunction [15]. Furthermore, autophagy is closely associated with inflammation [16, 17]. Loss of the autophagy protein Atg16L1 increases IL-1beta production induced by endotoxin [18]. Dysfunction of autophagy related 16-like 1 (ATG16L1) triggers chronic intestinal inflammation [19].

Interestingly, MP administration causing plasma zinc decreasing in human was reported [20–22]. Rekers recently found that MP regulates intracellular concentrations of zinc through influencing MT-1 expression [23]. Nowadays zinc is increasingly recognized as an ionic messenger or a neurotransmitter more than a micronutrient [24]. In our previous study, we demonstrated that spinal cord zinc changes after SCI. In the current study, we suppose that the neuronal protective role of MP may relate to the activation of autophagy through the changes of zinc, as an ionic messenger.

## 2. Methods and Materials

### 2.1. Animals and Drug Administration

Adult male Sprague-Dawley rats (220 ± 20.0 g, aged 2-3 months) were purchased from Capital Medical University (Beijing, China), and the study was approved by the Animal Care and Use Committee of Liaoning Medical University. Five animals were used in each experiment group at each time point. The animals were housed in individual cages with 12 h light/dark schedule, relative humidity of 50%, controlled temperature (24 ± 1°C), and free access to water and food before and after surgery. Rats were randomly and evenly divided into two groups: SCI-only group and SCI + MP group. Compared to SCI-only group, rats of SCI + MP group received not only SCI, but also injection of methylprednisolone (MP, 30 mg/kg, i.v., Sigma-Aldrich, St. Louis, MO, USA) once a day in first week. And in each group, subgroups were set up as normal animal (without injury), 1 hour (1 h), 6 hours (6 h), 12 hours (12 h), 18 hours (18 h), 1 day (1 d), 3 days (3 d), 7 days (7 d), 14 days (14 d), 21 days (21 d), and 60 days (60 d) after SCI.

### 2.2. Acute Spinal Cord Injury Model

Following 10% chloral hydrate (2 mL/kg, i.p.) anesthesia, rats were positioned on a platform, with continuous rectal temperature monitored and maintained at 37.0 ± 0.5°C by a heating pad. Laminectomy was carried out at the level of T10 to expose the intact dorsal cord surface. Then a contusion was induced by a self-made electromagnetic programmed weight-drop device in the spinal cord corresponding to the T10 spinous process, centering at the posterior median spinal vessels. The striking force was 25 × 3 g∗cm: the iron stick was 25 g in weight and 3 cm in bottom diameter, the dropping distance was 3 cm, and the time of contact with the dura mater was 0.1 s. After operation, the wound was sterilely closed.

### 2.3. Primary Microglia Cell Cultures

Spinal cord tissues from postnatal day 1 SD rat were collected and mechanically fragmented, then digested with 2.5 mg/mL trypsin-EDTA buffered with 10 mM HEPES (GIBCO) for 12 min at 37°C, and finally mechanically dissociated. Cells were planted on poly-D-lysine (30–70 kDa, Sigma-Aldrich, St. Louis, MO, USA) coated dishes in DMEM medium, 10% FBS, and 1% penicillin/streptomycin solution (GIBCO). After 10 days, microglia were dislocated by the addition of 12 mM lidocaine. Isolated microglia were planted on poly-D-lysine coated plates at a density of 1.5∗10^∗5^ cells/mL in DMEM medium (10% FBS, 1% penicillin/streptomycin solution).

### 2.4. Behavioral Test

At different time points (normal, 3 d, 7 d, 21 d, and 60 d) of each group, behavioral testing was analyzed as described previously [25, 26]. All experiments were performed in a double-blind manner.

### 2.5. Nissl Stain

Spinal cord sections of rats were prepared as previously described [27]. Every tenth section was collected and stained with cresyl violet in each group.

### 2.6. Immunohistochemistry

Spinal cord sections of rats were prepared as previously described [27]. The following antibodies were applied: goat anti-Iba-1 (ionized calcium binding adaptor molecule 1, microglia-specific marker) antibody (4 *μ*g/mL; Abcam Cambridge, UK). The following secondary antibodies were applied: rabbit anti-goat IgG secondary antibody (1 : 500; Origene).

### 2.7. RNA Extraction and RT-PCR Analysis

Spinal cord tissues were collected from rats in each group, total mRNA were generated as previously described, and real-time PCR was analyzed as previously described by using an Applied Biosystems 7500 Real-Time PCR System (Foster City, CA, USA) [28]. In brief, according to our previous protocol, we also use TRIzol RNA isolation reagent to purify total cellular RNA and GAPDH (Chemicon International Temecula, CA, USA) as the internal reference. The iNOS (inducible nitric oxide synthase), IL-6 (interleukin-6), IL-10 (interleukin-10), Beclin-1, LC-3B (microtubule-associated protein 1 light chain 3B), and ZIP8 (zinc transport SLC39A8) primer sequences were selected by using a Lasergene (DNA Star Inc., WI, USA) program.

### 2.8. Atomic Absorption Spectrometry

Spinal cord sections of rats at injured site were collected to be weighed, and we recorded the weight (about 0.5 g–1 g). Then specimens were moved to digestion tank and digested by automatic digestion apparatus (ST-60, Polytech, China) as in the following steps: (1) adding 100% HNO_3_ 4 mL and 100% HClO_4_ 1 mL, respectively; (2) vibrating at 100% speed for 1 minute; (3) heating at 100°C for 30 minutes; (4) vibrating at 100% speed for 1 minute; (5) heating at 180°C for 30 minutes; (6) cooling down for 30 minutes; and (7) diluting volumes to 20 mL with 1% HNO_3_. Then solutions were measured with atomic absorption spectrometry (PE AA800, Perkinelmer, USA) for zinc. Venous blood samples (4 mL) of rats were collected for analysis of zinc, and after centrifugation, serum was stored at −20°C until analysis. The samples were thawed at 37°C and diluted to 20 mL with 1% HNO_3_. Then samples were detected by flame atomic absorption spectrometry.

### 2.9. Immunofluorescence Staining

In vitro, lipopolysaccharide (LPS, 100 ng/mL, Sigma-Aldrich, St. Louis, MO, USA) was added into the primary cultured microglia; at the same time MP (10 *μ*M) was added into the LPS inflammation model. The details of staining were as previously described [29]. The following primary antibodies were used, based on differing targets: goat anti-Iba-1 antibody (4 *μ*g/mL; Abcam Cambridge, UK). The following secondary antibodies were applied: FITC donkey anti-goat IgG secondary antibody (1 : 500; Abcam Cambridge, UK).

### 2.10. Western Blot

Western blot was performed by using a standard protocol as previously described [29]. Samples were normalized to 1 mg/mL and the loading volume was 20 mL/well. The membranes were, respectively, incubated with rabbit polyclonal anti-LC-3B (1 : 500; Novus Biologicals), rabbit polyclonal Beclin-1 (1 : 1000; Abcam Cambridge, UK), rabbit polyclonal ZIP8 (1 : 1000; Santa Cruz Biotechnology), rabbit polyclonal nuclear factor-kappa beta (NF-*κβ*) (1 : 1000, Abcam Cambridge, UK), rabbit polyclonal anti-iNOS antibody (1 : 1000, Abcam Cambridge, UK), and rabbit anti-*β*-actin (1 : 500; Santa Cruz Biotechnology). The bound antibodies were detected using goat anti-rabbit IgG-HRP antibody (1 : 1000; Abcam Cambridge, UK). The protein bands were visualized by an ECL detection system (Pierce Chemical, Rockford, IL, USA) and quantified by Image J software (NIH, Bethesda, MD).

### 2.11. ELISA

Spinal cord tissues were collected from rats and dissected and homogenized in RIPA buffer. IL-6, IL-10, and TNF-*α* (tumor necrosis factor-*α*) (both Origene) were measured using respective ELISA kit according to the manufacturer's instructions and analyzed by microplate reader (Dynex Technology, Chantilly, VA, USA).

### 2.12. Cell Proliferation Analysis

Cells were planted on 96-well plates at 4000 cells per well. Different drug administration (nothing, MP (10 *μ*M), LPS (100 ng/mL), LPS (100 ng/mL) + 3-MA (5 *μ*M, Sigma-Aldrich, St. Louis, MO, USA), LPS (100 ng/mL) + rapamycin (200 nM, Sigma-Aldrich, St. Louis, MO, USA), LPS (100 ng/mL) + MP (10 *μ*M), LPS (100 ng/mL) + MP (10 *μ*M) + 3-MA (5 *μ*M), and LPS (100 ng/mL) + MP (10 *μ*M) + TPEN (tetrakis (2-pyridulmethy-1) ethylenediamine, 1 *μ*M) (Santa Cruz Biotechnology)) were added to microglia cultures. A day later, cell proliferative activity was assessed by 3-(4,5-dimethylthiazol-2-yl)-2,5-diphenyltetrazolium bromide (MTT) assay (Sigma-Aldrich) according to the instructions of the manufacturer. Absorbance was measured at a test wavelength of 570 nm and a reference wavelength of 630 nm for each well using a microplate reader (Dynex Technology, Chantilly, VA, USA).

### 2.13. Cellular Zinc Stain

Primary cultured microglia were stained with 5 *μ*M FluoZin-3-AM (Invitrogen) in culture media for 15 min in a humidified CO_2_ incubator, imaged by fluorescence microscope, and analyzed by microplate reader (Dynex Technology, Chantilly, VA, USA) according to the manufacturer's instructions.

### 2.14. Statistical Analysis

Data are expressed as the mean ± SEM or SD. Statistical evaluation of the data was performed using one-way analysis of variance (ANOVA) and Dunnett's post hoc test. *P* values < 0.05 were considered statistically significant.

## 3. Result

### 3.1. MP Has a Neuroprotective Effect on SCI

**Figure 1:**
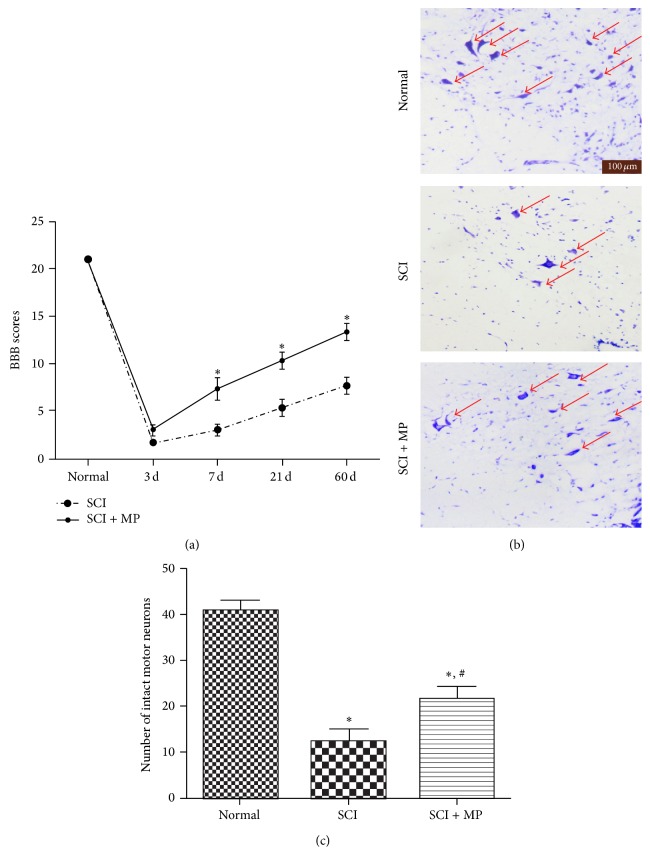
MP promoted neurologic motor function and histologic assessment in SCI rat. (a) BBB scores of rats in SCI-only group and MP group at each time point (3 d, 7 d, 21 d, and 60 d) after injury. Data were expressed as mean ± SD (*n* = 8 for each group); (b) representative sections of spinal cords in the ventral horn of gray matter stained with cresyl violet at 72 h after injury. Normal neurons exhibited a fine cytoplasm with Nissl substance (arrows); scale bar = 100 *μ*m; (c) quantitative motor neurons counts in the ventral gray matter at 72 h after injury. Data were expressed as mean ± SEM (*n* = 3 for each group). ^*∗*^ *P* < 0.05 versus normal group; ^#^ *P* < 0.05 versus SCI group.

To evaluate the effect of MP on the recovery of SCI, BBB scores firstly were assessed in SCI group and SCI + MP group at different time points (3 d, 7 d, 21 d, and 60 d) after injury. As shown in Figure 1(a), the averages of the total BBB scores were significantly lower in SCI group than SCI + MP group since 7 d after injuries. And especially 60 days after contusion, MP treatment increased BBB scores to around 11 compared to 7 of SCI group, indicating that locomotor activity was significantly promoted by MP. Furthermore, the effect of MP on the numbers of motor neurons in spinal cord was investigated using Nissl staining at 3 days after SCI (Figure 1(b)), and average motoneuron counts per section in thoracic spinal cord of rat were calculated (Figure 1(c)). SCI group showed extensive loss of large anterior horn cells. In contrast, motor neurons of the anterior horns were significantly reserved in rats treated with MP.

### 3.2. MP Reduced Microglia Activation Maybe via Beclin-1 Dependent Autophagic Cell Death after Spinal Cord Injury

**Figure 2:**
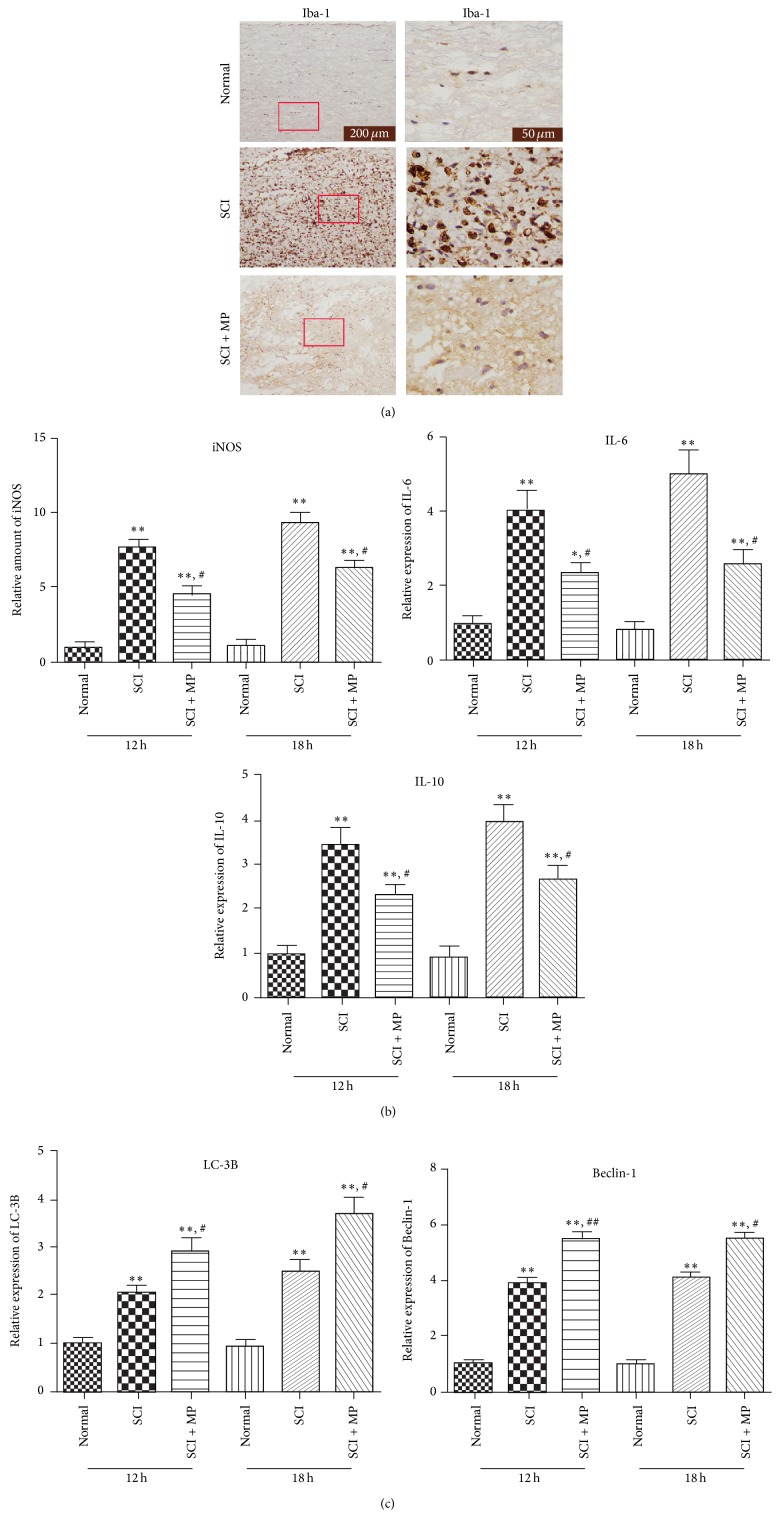
MP reduced the activation of microglia in SCI rats. After 12 hours and 18 hours treatment, spinal cord was collected for histological analysis, ELISA and RT-PCR. (a) Immonoexpression of Iba1 (brown) in spinal cord at 12 h and 18 h. Scale bar = 200 *μ*m, 50 *μ*m; (b), (c) Spinal cord around lesion was subjected to examine the expression of iNOS, IL-6, IL-10, LC-3B and Beclin-1 by RT-PCR. Data were expressed as mean ± SD (*n* = 5 for each group). ^*∗*^ *P* < 0.05, ^*∗∗*^ *P* < 0.01 versus normal group; ^#^ *P* < 0.05, ^##^ *P* < 0.05 versus SCI group.

In order to examine whether microglia activation was inhibited by MP, the representative protein Iba-1 of microglia/macrophages was assessed by immunohistochemistry. Twelve hours after MP administration, number of activated microglia/macrophages, stained by Iba-1, was apparently reduced, compared to a mass of expression in SCI-only group (Figure 2(a)). Furthermore, other relevant markers associated with activation of microglia were then tested by RT-PCR. Levels of iNOS, IL-6, and IL-10 in SCI + MP group at 12 h and 18 h were lower than those in SCI group (Figure 2(b)). And we supposed whether autophagic cell death resulted in the decreased number of microglia; the reprehensive proteins of autophagy were assessed by western blot. The levels of Beclin-1 and LC-3B, as we expected, were significantly increased in SCI + MP group compared to SCI-only group (Figure 2(c)).

### 3.3. MP Increases the Level of Zinc and ZIP8

**Figure 3:**
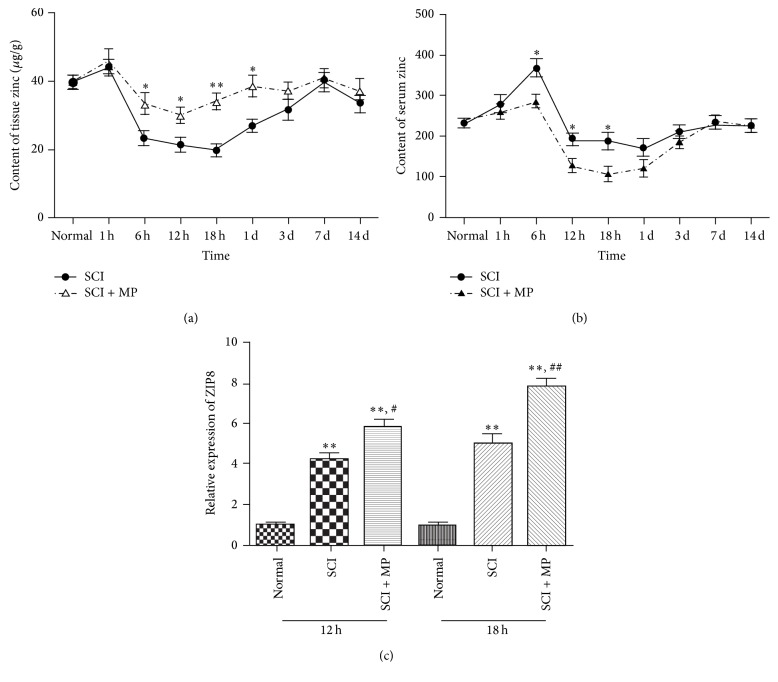
Effects of MP on zinc level. Spinal cords around lesions (a) and serum (b) from different time points (1 h, 6 h, 12 h, 18 h, 1 d, 3 d, 7 d, and 14 d) were used to measure the level of zinc by atomic absorption spectrometry; (c) expression of ZIP8 was detected by RT-PCR at 12 h and 18 h. Data were expressed as mean ± SEM (*n* = 3 for each group). ^*∗*^ *P* < 0.05, ^*∗∗*^ *P* < 0.01 versus control group; ^#^ *P* < 0.05, ^##^ *P* < 0.01 versus LPS group.

In order to examine whether tissue zinc level changed after MP administration in SCI rat, atomic absorption spectrometry was used to quantify the content of zinc elements. As shown in Figure 3(a), tissue zinc decreased from 40 *μ*g/g to 22 *μ*g/g in six hours after SCI and returned to baseline zinc level seven days after withdrawal. MP significantly inhibited the decreases of tissue zinc from 6 h to 1 d. The results suggest that MP SCI could revert the tissue zinc deficiency that existed in SCI rats. So we had to assess changes of serum zinc by atomic absorption spectrometry, corresponding to tissue zinc. Serum zinc level was significantly increased 6 hours later in SCI group. However, it was significantly decreased at 12 h and 18 h (180 um/mL) and the level of zinc returned to baseline zinc level from day 7 after SCI. Serum zinc levels in SCI + MP group were lower than those in SCI groups at different time points (6 h, 12 h, and 18 h) (Figure 3(b)). These results suggest that MP could inhibit the increased serum zinc in SCI rats. To further test zinc transporter we measured ZIP6, ZIP8, and ZIP14 by RT-PCR. Comparing SCI group with SCI + MP group, only ZIP8 has shown significant difference, which was found to be upregulated in SCI + MP groups compared to SCI groups (Figure 3(c)).

### 3.4. Microglia Activation Is Inhibited by MP In Vitro

**Figure 4:**
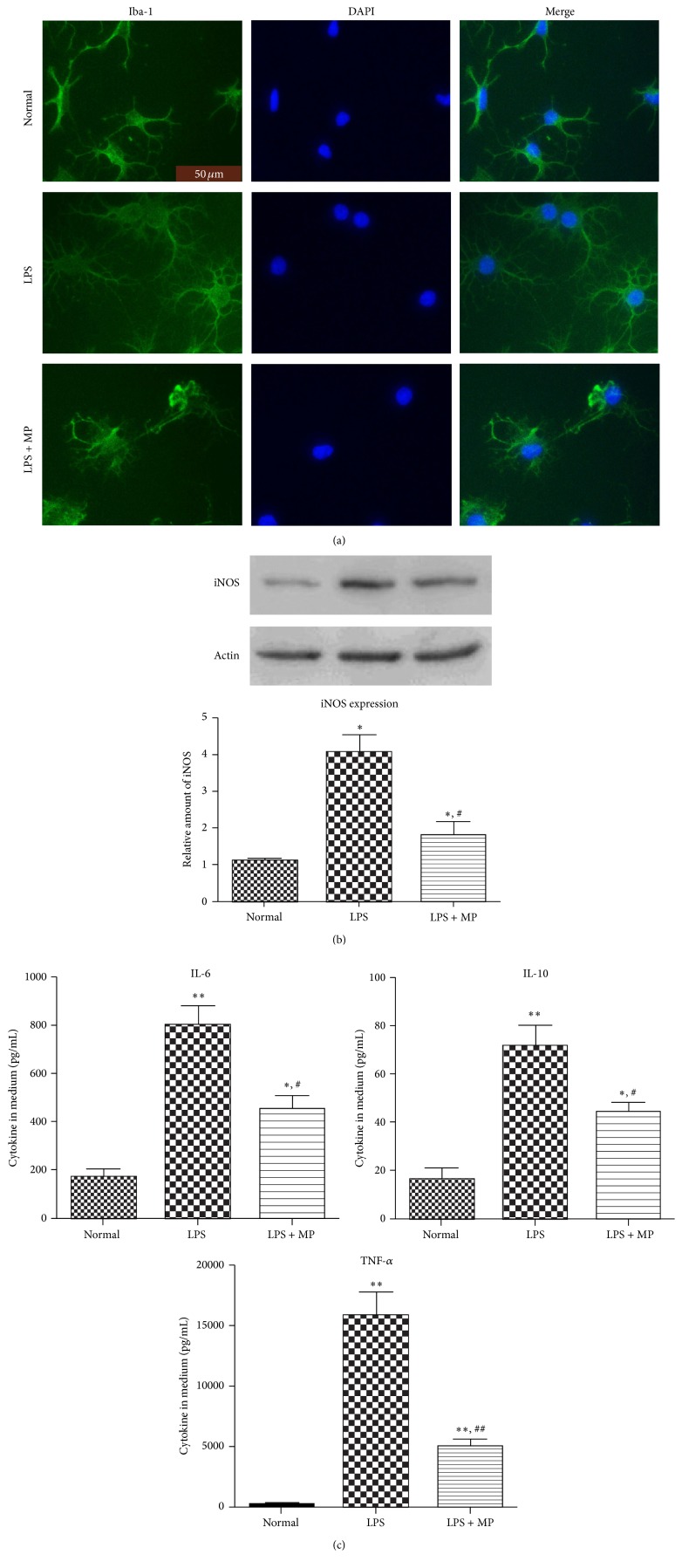
Effects of MP on microglia activation and secretion of inflammatory cytokines. (a) The expression of Iba-1 of microglia. After 12 hours of treatment, immunofluorescence staining was performed to assess expression of Iba-1 (Iba-1 green; DAPI blue); scale bar = 50 *μ*m. (b) After 12 hours of incubation, the expression of protein iNOS in microglia was assessed in LPS group, LPS + MP group, and normal group by western blot. (c) Cytokine levels in culture medium. After 12 hours of treatment, the culture media were collected and expression of IL-6, IL-10, and TNF-*α* was detected by ELISA kit. Data were expressed as mean ± SD (*n* = 5 for each group). ^*∗*^ *P* < 0.05, ^*∗∗*^ *P* < 0.01 versus normal group; ^#^ *P* < 0.05 versus LPS group.

To confirm whether MP could inhibit microglia activation in vitro, by MTT, we first choose a proper concentration of MP on microglia, not influencing cell state and viability after lasting 24 h incubation. The result shows that at concentration of 10 um MP has no influence on cell state and viability of microglia. Then we visualized morphology of microglia in each group by immunofluorescence staining of Iba-1. The pictures revealed significant differences in morphology between the groups. In normal group, nonactivated microglia showed long, thin, branched processes. And in LPS group (100 ng/mL LPS alone), microglia undergoing moderate activation had larger somata, thickened proximal processes, and retracted distal processes. Interestingly, in LPS + MP group (100 ng/mL LPS plus 10 um MP), we found that microglia, expressing disordered and retracted distal processes, were in poor state, with large somata and nucleus, known as morphology of autophagic cell death (Figure 4(a)). To further confirm effects of MP on inhibiting microglia activation, we used western blot to assess iNOS protein expressions level, after exposing microglia to MP (10 *μ*M) in LPS induced activation of microglia lasting 12 h, at which the iNOS expression level is the strongest following LPS treatment [30]. The results indicate that MP significantly inhibited LPS induced increase of iNOS proteins (Figure 4(b)). Finally, in the same conditions as described above, we assessed TNF-*α*, IL-6, and IL-10 cytokine secretion levels in microglia culture medium by ELISA (Figure 4(c)). According to our prospects, protein expressions of cytokine secretion levels of these cytokines were downregulated in varying degrees.

### 3.5. Beclin-1 Dependent Autophagic Cell Death Was Induced by MP When Microglia Were under Stress In Vitro

**Figure 5:**
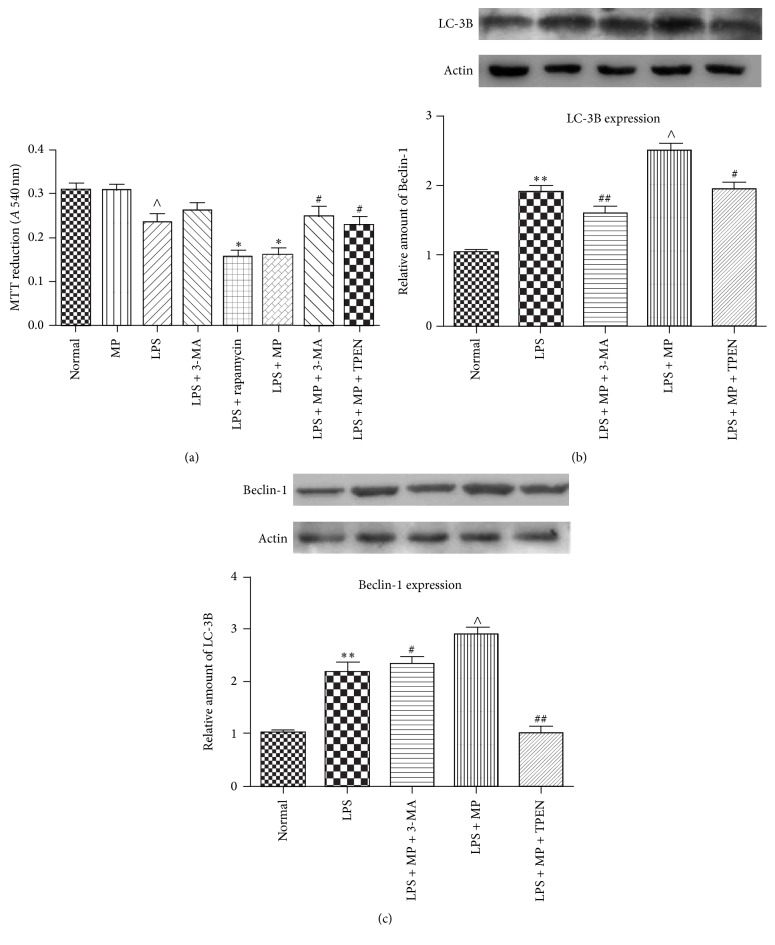
MP induced autophagic cell death of microglia in vitro in LPS induced inflammation model. (a) After 12 hours of incubation, cell viability of microglia was measured in normal group, LPS group, LPS + 3-MA group, LPS + rapamycin group, LPS + MP group, LPS + MP + 3-MA group, and LPS + MP + TPEN group by MTT. MTT reductions were expressed as mean ± SD (*n* = 5 for each group). ^*∗*^ *P* < 0.05 versus LPS group; ^#^ *P* < 0.05 versus LPS + MP group; ^∧^ *P* < 0.05 versus normal group. (b), (c) After incubation as described above, the expression of LC-3B (b) and Beclin-1 (c) in microglia was assessed by western blot in normal group, LPS group, LPS + MP + 3-MA group, LPS + MP group, and LPS + MP + TPEN group. Relative amounts were expressed as mean ± SD (*n* = 5 for each group). ^*∗∗*^ *P* < 0.01 versus normal group; ^#^ *P* < 0.05, ^##^ *P* < 0.01 versus LPS + MP group; ^∧^ *P* < 0.05 versus normal group.

In vitro, we assessed effect of MP on cell viability of microglia by MTT. The results showed that MP did not influence cell viability of microglia when cell state was normal, that MP significantly enhanced the death rate when microglia were under stress, that rapamycin also enhanced the death rate when microglia were under the same stress, and that the death rate of LPS + MP groups was suppressed by 3-MA or TPEN (Figure 5(a)). Then we assessed autophagy through detected LC-3B protein by western blot, which is often used to detect the levels of autophagy. There appeared to be an upregulation of LC-3B in LPS groups. MP + LPS treatment had a more increasing level of LC-3B than LPS groups, which was suppressed following treatment with 3-MA or TPEN (Figure 5(b)). To further evaluate whether the effect of MP on autophagy depended on Beclin-1, Beclin-1 expression of each group was detected via western blot analysis. As shown in Figure 5(c), the expression of Beclin-1 was almost consistent with LC-3B protein expression levels. All of these results suggest that Beclin-1 dependent autophagic cell death was induced by MP.

### 3.6. MP Upregulates Cellular Labile Zinc Level and ZIP8 and Inhibits NF-*κβ*

**Figure 6:**
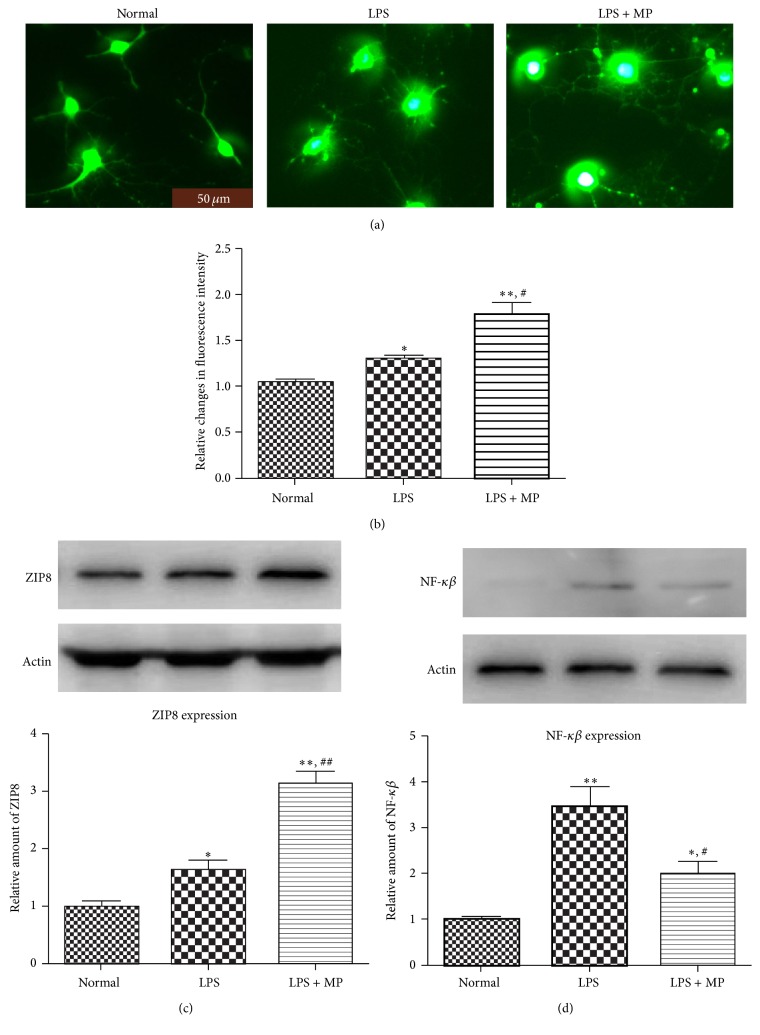
Increasing cellular zinc inhibited NF-*κβ* in microglia. (a) After 12 hours of treatment, microglia were stained with FluoZin-3-AM in normal group, LPS group, and LPS + MP group. Scale bar = 50 *μ*m. (b) The relative fluorescence intensity of zinc. Data were expressed as mean ± SD (*n* = 5 for each group). ^*∗*^ *P* < 0.05, ^*∗∗*^ *P* < 0.01 versus normal group; ^#^ *P* < 0.05, versus LPS group. (c) After the same disposition as described above, the membrane protein of microglia was collected and the expression of ZIP8 was detected by western blot. Relative density of ZIP8 was analyzed. Data were expressed as mean ± SEM (*n* = 3 for each group). (d) After the same disposition as described above, the expression of NF-*κβ* in microglia was observed by western blot. Data were expressed as mean ± SD (*n* = 5 for each group). ^*∗*^ *P* < 0.05, ^*∗∗*^ *P* < 0.01 versus normal group; ^#^ *P* < 0.05, ^##^ *P* < 0.01 versus LPS group.

We examined cellular labile zinc changes after microglia were activated. After cells were disposed in the same way as described above, microglia were stained with FluoZin-3-AM, a zinc-specific fluorescent dye. Consistent with our prospects, imaging showed that the concentrations of cellular labile zinc increased in activated microglia after LPS treatment, and that MP administration caused a further increase of concentrations obviously (Figures 6(a) and 6(b)). To test ZIP8 changes, the membrane protein was collected and the expression of ZIP8 was detected by western blot. The results were in accord with the variations of cellular zinc we had found (Figure 6(c)). Recently NF-*κβ* was reported to be a major inhibitory target of zinc [31, 32]. Hence the protein of NF-*κβ* in microglia was detected by western blot. The results suggest that LPS induced upregulation of NF-*κβ* is significantly suppressed by MP (Figure 6(d)).

## 4. Discussion

Indeed, microglia activation is a feature of secondary mechanisms of SCI; some of the properties of that are beneficial, for example, protecting spinal cord from infections and confining injury regions [33, 34]. However, the excessive and prolonged activation of microglia causes significant damage to neurologic recovery in SCI [35]. A pharmacological intervention on the microglia activation could be a promising therapeutic target. Thus, our result is noteworthy that MP administration obviously reduced density of microglia in injured spinal cord (Figure 2(a)), which was correlated with decreased axonal injury. And in our in vitro studies, we now report that cell viability of microglia was significantly reduced by MP administration when microglia were undergoing activation (Figure 5(a)). Besides, we found that MP + LPS administration resulted in morphology manifestations of disordered and retracted distal processes and in poor state with large somata and nucleus (Figure 4(a)), known as morphology manifestation of autophagic cell death [36]. These results suggest that MP could directly inhibit excessive microglia activation and that the inhibition may be related to autophagic cell death of microglia. Besides, microglia activation is mainly associated with increased iNOS and proinflammatory cytokines, such as TNF-*α*, IL-6, and IL-10, which not only are inflammatory markers associated with microglia/macrophages, but also could produce a neuroinflammatory environment [37] and play critical roles in the pathogenesis of SCI [38]. We detected a significant downregulation of proinflammatory molecules iNOS, IL-6, and IL-10 by MP administration following SCI (Figure 2(b)). And in our in vitro studies, MP decreased the protein expression of iNOS and IL-6, IL-10, and TNF-*α* levels in culture medium in diverse degrees (Figures 4(b) and 4(c)). These results further certify an inhibitory effect of MP administration on the microglia activation and attendant inflammatory molecule production.

As described above, MP may cause autophagic cell death of microglia to inhibit microglia activation. Indeed, whether autophagy is good or bad is argued [39]. Autophagy was upregulated in amyotrophic lateral sclerosis, maybe causing autophagic cell death, which may increase the loss of motor neurons [40]. By contrast, induction of autophagy can induce neuroprotective effects after SCI via inhibition of apoptosis [41]. In the current study, autophagic cell death that occurred in microglia is supposed to be good for SCI, for its inhibition of microglia activation. ATG8/LC3, microtubule-associated protein 1 light chain 3, exists in two forms, as LC-3A and LC-3B. And LC-3B or LC-3B/LC-3A ratio is positive correlation with autophagy [16]. It is also known that autophagy could be divided into Beclin-1 dependent and nondependent according to the diverse mechanisms and that Beclin-1 is also associated with the induction of autophagic cell death [42]. Coinciding with our supposition, we have found that expression of both LC-3B and Beclin-1 was upregulated by MP after SCI (Figure 2(c)), which indicated that MP strengthened Beclin-1 dependent autophagy after SCI. But whether autophagy occurs in microglia, or in other cells of spinal cord except microglia? In in vitro study, we focus on effect of MP on autophagy in microglia. We found that LPS induced increases of both LC-3B and Beclin-1 were significantly upregulated by MP (Figures 5(b) and 5(c)) and that 3-MA, a powerful inhibitor of autophagy now widely used [43], reverted the enhancement caused by MP, which verified our supposition. Actually, the cell viability was reduced by rapamycin in LPS model; comparing LPS group and LPS + rapamycin group, we supposed that autophagic cell death induced by rapamycin may be the main reason of the enhanced cell death and that autophagic cell death later could be induced in LPS model (Figure 5(a)). Furthermore, combining with the results that MP reduced cell viability, compared LPS + MP group to LPS group, and that 3-MA significantly reverted the reduced cell viability of MP, compared LPS + MP group to LPS + MP + 3-MA group, we supposed that autophagic cell death may be main reason of MP reduced cell viability in LPS model (Figure 5(a)). Interestingly, we also have found that there was no statistical difference between LPS + 3-MA group and LPS + 3-MA + MP group; we supposed that 3-MA maybe mostly inhibit the Beclin-1 depended autophagy pathway in LPS + 3-MA + MP group, so MP did not reduce cell viability as like the MP did in LPS + MP group, compared to LPS group (Figure 5(a)). Besides, comparing LPS group and LPS + 3-MA group, there was also no statistical difference, which indicated that LPS induced cell death was not mainly through autophagic cell death (Figure 5(a)). So we suggested that when undergoing stress, autophagic signaling of microglia is activated but the level of autophagy is not enough to cause autophagic cell death and that MP administration could then strengthen the level of autophagy, finally causing autophagic cell death of microglia.

Zinc is greatly associated with autophagy; endogenous zinc plays key role as a trigger in autophagy [44, 45]. In keeping with previous results that MP administration results in a decrease of serum zinc in human [20–22], our findings extend these previous reports by that MP caused a redistribution of zinc from serum to injured spinal cord, resulting in an obvious increase of cellular zinc in injured spinal cord (Figures 3(a) and 3(b)). The importance of zinc redistribution is emphasized in many diseases [46]. Free zinc within cells recently has been ascribed status of neurotransmitter functions, emphasizing the roles of zinc in biology [47]. Hence, combining our current studies, we supposed that effect of MP on autophagy may mainly depend on zinc. Interestingly, at the beginning of SCI, there was an increase of serum zinc in both SCI-only and SCI + MP groups (Figure 3(b)); we suppose that SCI may cause loss of zinc from damage cells or mesenchyme into blood. Besides, after one week, tissue zinc was going to decrease (Figure 3(a)), which suggests that there was a deficiency of zinc after SCI, coinciding with our previous results. Due to the fact that zinc was redistributed by MP to injured spinal cord, we supposed ZIP6, ZIP8, or ZIP14 protein would change in spinal cord tissue, which mainly had a function of transporting zinc inside cells. And we have found that ZIP8 changed greatly and this was coinciding with the variation of serum and tissue zinc (Figure 3(c)). Actually ZIP8 expression is critical in the protection of zinc against tissues damage. It participates in protection of zinc against tnf-alfa induced damage [48], deregulation of proinflammatory responses [49], and so on. Combining these results with our in vitro result that MP caused a further increase of ZIP8 (Figure 6(c)), we further suggested that the increased cellular free zinc of microglia after SCI may mainly come from outside, not from inside as we supposed. As described above, zinc was supposed to be main participant in autophagic cell death of microglia; we test the cellular labile zinc intensity of microglia in vitro. We found that LPS induced an increase in concentrations of cellular labile zinc and that MP administration obviously caused a further increase, maybe mainly through ZIP8. Furthermore, with our results that TPEN, a zinc chelator, reverted the reduced cell viability caused by MP (Figure 5(a)) and that TPEN reduced MP induced enhancement of autophagy (Figures 5(b) and 5(c)), we suggested that increasing concentrations of cellular labile zinc has a key role in autophagic cell death of microglia caused by MP. Besides, recently NF-*κβ* was reported to be a major inhibitory target of zinc [31, 32]. What is more important is that Ming-Jie Liu reports that cellular zinc increasing was confirmed to cause downregulation of NF-*κβ*, which was the result of increased ZIP8 expression [49]. According to previous results, we also found that NF-*κβ* was downregulated after MP treatment (Figure 6(d)). At the same time, currently, there are also advances which have demonstrated that activation of NF-*κβ* could inhibit autophagy [50]. Hence, we supposed that MP caused concentrations of cellular labile zinc to increase, which may downregulate expression of NF-*κβ*, which finally results in upregulating autophagy of microglia. More studies should be done to confirm these suppositions.

Both BBB scores (Figure 1(a)) and motor neurons numbers of the anterior horns (Figure 1(c)) were significantly higher in rats treated with MP than the SCI-only rats. Combined with Nissl staining results (Figure 1(b)), these results indicated that autophagic cell death of microglia induced by MP administration protected neuronal locomotor function and enhanced the behavioral recovery of rats after SCI.

## Abbreviations

SCI: Spinal cord injury
BBB scores: Basso, Beattie, and Bresnahan scores
MP: Methylprednisolone
Iba-1: Ionized calcium binding adaptor molecule-1
iNOS: Inducible nitric oxide synthase
IL-6: Interleukin-6
IL-10: Interleukin-10
TNF-*α*: Tumor necrosis factor alpha
NF-*κβ*: Nuclear factor-kappa beta
LC-3B: Microtubule-associated protein 1 light chain-3B
ZIP8: Zinc transport SLC39A8
LPS: Lipopolysaccharide
TPEN: Tetrakis(2-pyridulmethy-1)ethylenediamine.
